# Bronchoscopy-Guided Intervention Therapy With Extracorporeal Membrane Oxygenation Support for Relapsing Polychondritis With Severe Tracheobronchomalacia: A Case Report and Literature Review

**DOI:** 10.3389/fmed.2021.695505

**Published:** 2021-11-23

**Authors:** Pengcheng Zhou, Bing Fu, Chuantao Zhang, Keling Chen, Qianming Xia, Wenjun Tang, Wei Yu, Wenhui Huang

**Affiliations:** ^1^Department of Respiratory Medicine, Hospital of Chengdu University of Traditional Chinese Medicine, Chengdu, China; ^2^Department of Cardiothoracic Surgery, Hospital of Chengdu University of Traditional Chinese Medicine, Chengdu, China; ^3^Department of Respiratory Medicine, The Aviation Industry Corporation of China (AVIC) 363 Hospital, Chengdu, China; ^4^Clinical Medical School, Chengdu University of Traditional Chinese Medicine, Chengdu, China

**Keywords:** extracorporeal membrane oxygenation, relapsing polychondritis, tracheobronchomalacia, severe airway stenosis, bronchoscopy, interventional therapy

## Abstract

Relapsing polychondritis is an immune disorder of unknown etiology involving multiple systems that is characterized by persistent inflammation and destruction of cartilage, including the ears, nose, costal, joint, and airways. Airway involvement caused by relapsing polychondritis is common, and tracheobronchomalacia is the most serious complication, which is life-threatening. Currently, the exact mechanism of relapsing polychondritis with tracheobronchomalacia is unknown. Although glucocorticoids and immunosuppressive agents are administered, failures often occur. Currently, bronchoscopy-guided intervention therapy used in tracheobronchomalacia caused by chronic obstructive pulmonary disease or other etiology has gradually increased, but bronchoscopy-guided intervention therapy with extracorporeal membrane oxygenation assist used in tracheobronchomalacia caused by relapsing polychondritis has not been reported. Here, we report a case of relapsing polychondritis with severe tracheobronchomalacia. Although drug therapy was provided and airway stent implantation was performed, the tracheal stenosis was further aggravated. Because conventional anesthesia and mechanical ventilation cannot meet the needs of bronchoscopy-guided intervention therapy or guarantee sufficient safety. The intervention treatment was performed with the support of extracorporeal membrane oxygenation, which was successfully completed without obvious complications. The symptoms were significantly improved, and the patient was discharged uneventfully.

## Introduction

Relapsing polychondritis (RP) is a rare immune disorder involving multiple systems, in which the cartilage is the main target organ. The literature shows that at least 50% of RP cases involve the airway cartilage, among which tracheobronchomalacia (TBM) is the most serious complication (1). Currently, the mechanism of TBM caused by RP remains unclear; there is a lack of effective therapeutic drugs and the prognosis is extremely poor. Although glucocorticoids and immunosuppressants are often empirically recommended as first-line treatments, their clinical efficacy is often unsatisfactory. Despite the gradual increase in reports of new technologies such as stents and tracheobronchoplasty applied to TBM caused by chronic obstructive pulmonary disease (COPD) or other etiologies (2, 3), bronchoscopy-guided interventional therapy with extracorporeal membrane oxygenation (ECMO) support used in TBM caused by RP has not been reported. Here, we report a case of RP with severe TBM. Although glucocorticoids were administered according to the guidelines, the condition continued to worsen. Subsequently, the patient's symptoms and lung function significantly improved after the airway stent was implanted. However, as the disease progressed, severe stenosis appeared again from the subglottis to the upper segment of the tracheal stent. As conventional anesthesia and mechanical ventilation could not guarantee the safety of the operation, bronchoscopy-guided intervention was performed under the support of venous-venous (VV)-ECMO and was successful. After the treatment, the spirometry test showed improvement, and symptoms such as cough, shortness of breath, and hypoxia were significantly relieved.

## Case Presentation

**Figure 1:**
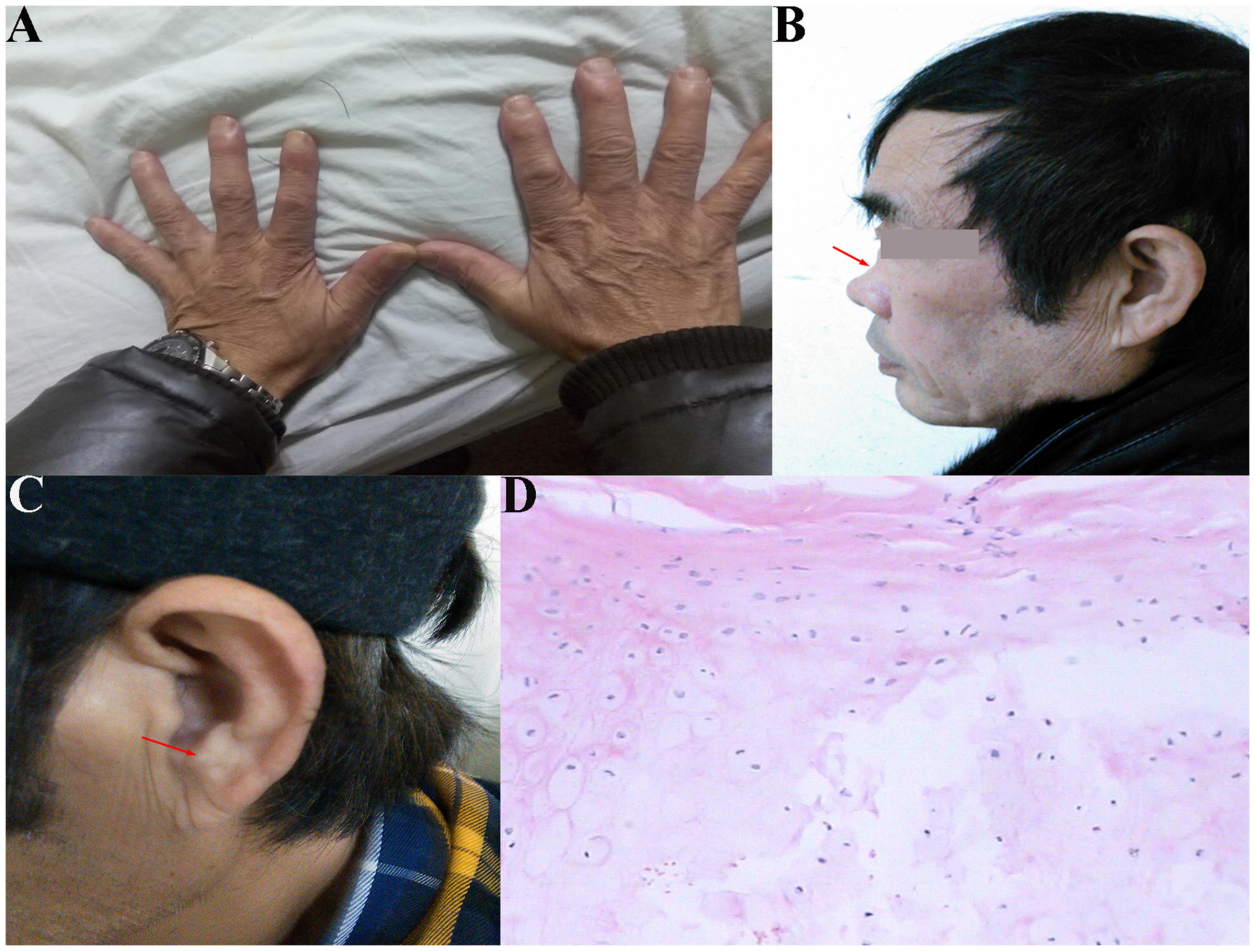
**(A)** Clubbing digits; **(B)** collapsed of the bridge of the nose (red arrow); **(C)** stunted auricles (red arrow); **(D)** pathology showed multiple necrosis of ear chondrocytes accompanied by inflammatory cell infiltration.

**Figure 2:**
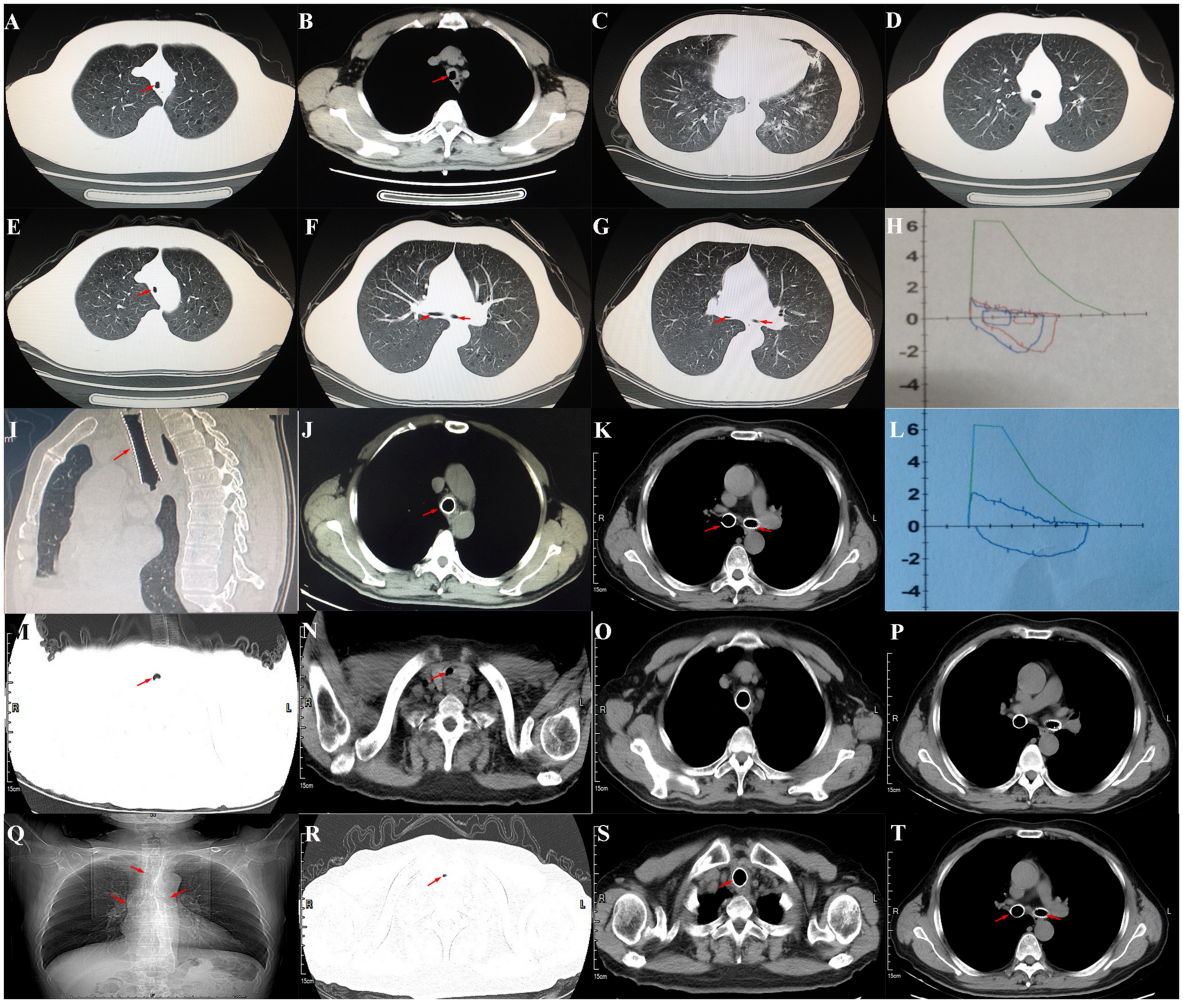
**(A)** Chest CT showed reduced tracheal lumen (red arrow); **(B)** thickened tracheal wall (red arrow); **(C)** emphysema and bilateral lung infections; **(D)** the lumen of trachea during the inspiratory phase (red arrow); **(E)** the lumen of trachea during the expiratory phase (red arrow); **(F)** the lumen of both mainstem bronchus during the inspiratory phase; **(G)** the lumen of both mainstem bronchus during the expiratory phase; **(H)** pulmonary function tests revealed severe mixed ventilatory dysfunction, which was mainly obstructive; **(I–K)** after the covered nickel-titanium memory alloy stent was implanted, the lumen was unobstructed (red arrow); **(L)** pulmonary function tests revealed moderately mixed ventilatory dysfunction, which was mainly obstructive; **(M,N)** chest CT showed that the lumen of subglottis to the upper of the stent was slightly narrowed (red arrow); **(O,P)** the stents were well fixed; **(R)** chest CT showed that the lumen of subglottis to the upper of the stent was severely narrowed; **(Q,S,T)** the stents were well fixed (red arrow).

**Figure 3:**
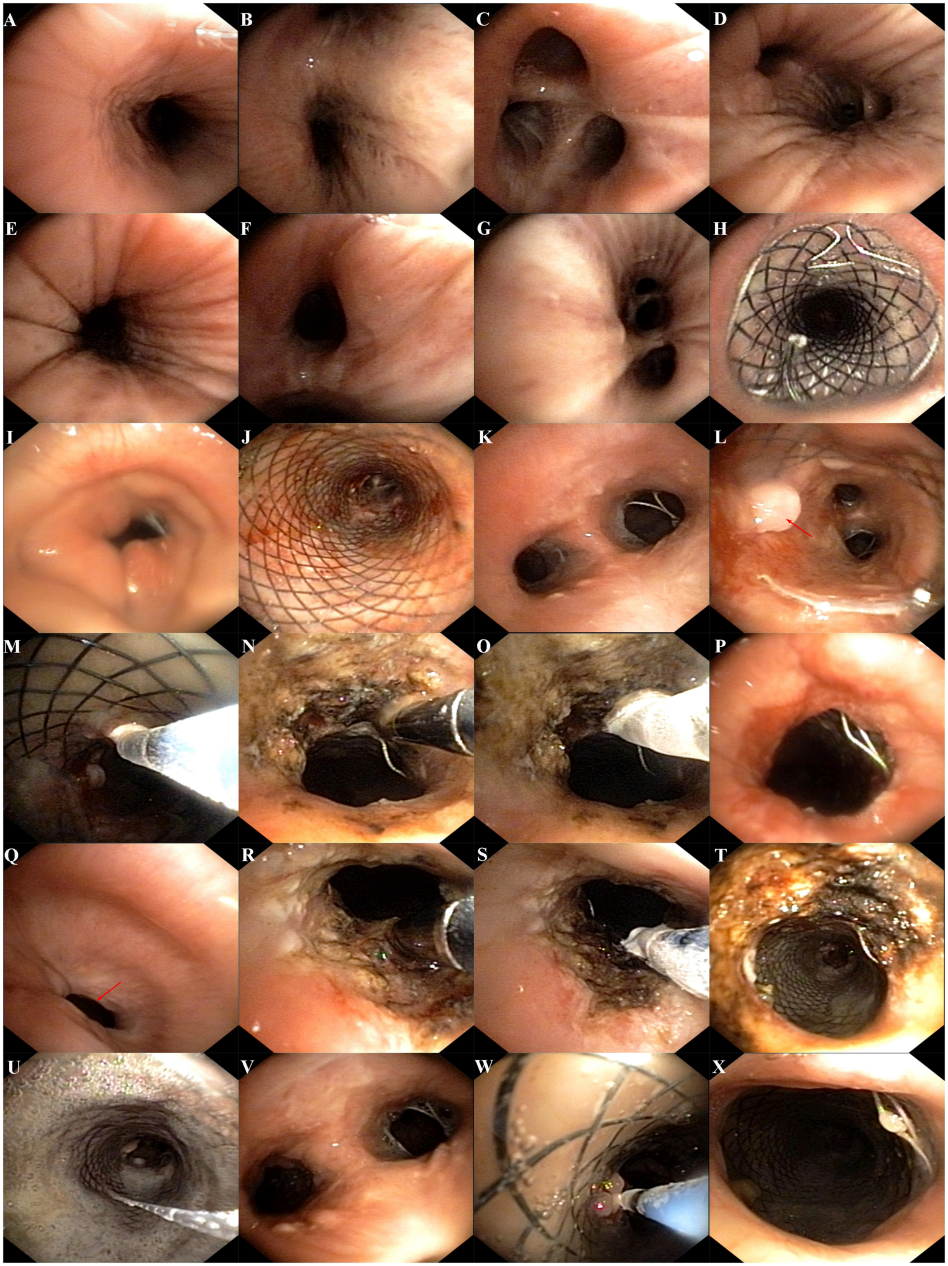
**(A–G)** Bronchoscopy showed that the mucosa of trachea and main bonchus were severely hyperemic and swollen, cartilage was disappeared, and the lumen was stenosis; **(H,J)** the stents were well fixed; **(I)** bronchoscopy showed that the lumen of subglottis to the upper of the stent was severe narrowed and accompanied by scar tissue; **(K,L)** bronchoscopy showed that the mucosa was severe swollen, cartilage was disappeared, and granulation hyperplasia can be found (red arrow); **(M)** CO_2_ cryotherapy of granulation tissue; **(N)** argon plasma coagulation was performed; **(O)** CO_2_ cryoablation was performed; **(P)** the airway lumen is obviously enlarged after therapy; **(Q)** bronchoscopy showed that the lumen of subglottis to the upper of the stent was severe narrowed and accompanied by scar tissue (red arrow); **(R,S)** bronchoscopy-guided argon plasma coagulation and CO_2_ cryoablation were performed; **(T)** the airway lumen is obviously enlarged after therapy; **(U,V)** granulation and a large amount of thick sputum blockage in the lumen; **(W)** CO_2_ cryotherapy of granulation tissue; **(X)** the airway lumen is further enlarged after therapy with ECMO assist.

The patient was a 60-year-old worker with repeated cough and dyspnea for ~10 years. The patient had a smoking history of 20 pack-years for 30 years and had chronic obstructive pulmonary disease and pulmonary bullae. Usually, these symptoms can be controlled using bronchodilators and inhaled corticosteroids; however, the dyspnea, cough, and sputum expectoration of the patient gradually worsened. The patient was admitted to our hospital for the first time due to sudden shortness of breath 1.5 years ago. Physical examination showed that the patient was thin, had clubbing digits, a slightly collapsed bridge of the nose, and had stunted auricles (Figures 1A–C). The chest was in a typical barrel shape, the intercostal space was widened, the breath sounds were lower, and wet rales can be heard in both lower lungs. Chest computed tomography (CT) showed reduced tracheal lumen, thickened tracheal wall, emphysema, and bilateral lung infections (Figures 2A–C). Pulmonary function tests revealed severe mixed ventilatory dysfunction, which was mainly obstructive, with a slight decrease in diffusion function (Figure 2H). Blood gas analysis suggested type 2 respiratory failure. Routine blood tests showed that white blood cells, neutrophils, and C-reactive protein were significantly elevated, suggesting an infection in the lungs. Although low-flow oxygen (2 L/min), antibiotics, glucocorticoids, and bronchodilators were administered, the patient's symptoms were not significantly relieved. A chest CT scan including the inspiratory and expiratory phases showed that the lumen of the trachea and main bronchus severely collapsed at the end of expiration compared with inspiration (Figures 2D–G). Subsequently, bronchoscopy showed that the mucosa of the trachea and main bronchi were severely hyperemic and swollen, and the cartilage had disappeared (Figures 3A–G). Although the lumen was normal during inhalation, the tracheal membrane protruded into the lumen during exhalation, resulting in the complete collapse of the lumen, and inability to eliminate secretions; these changes were not seen in the distal airway. A diagnosis of RP with TBM was highly suspected. Subsequently, a biopsy of the patient's auricular cartilage was performed. Pathology reports showed multiple necrotic chondrocytes accompanied by inflammatory cell infiltration (Figure 1D). Finally, the diagnosis was clear and consistent with our hypothesis. Subsequently, three nickel–titanium alloy coated memory stents were implanted in the trachea and bilateral main bronchus under local anesthesia (Figures 2I–K). Pulmonary function tests revealed moderate mixed ventilatory dysfunction, which was mainly obstructive, with a slight decrease in diffusion function (Figure 2L). Prednisone (1 mg/kg) was continued, and the patient was discharged. The patient was readmitted to our hospital for worsening dyspnea 1 year ago. Chest CT showed an unobstructed trachea and bilateral main bronchus, the stent was well-fixed, and the subglottis and upper part of the stent were slightly narrowed (Figures 2M–Q). Bronchoscopy showed that the lumen of the subglottis to the upper segment of the tracheal stent was narrow, the mucosa was severely swollen, cartilage had disappeared, and granulation hyperplasia was present (Figures 3H–L). The narrow lesion was significantly improved after bronchoscopy-guided argon plasma coagulation, and CO_2_ cryoablation was performed, which significantly relieved the patient's symptoms (Figures 3M–P). The patient was administered prednisone (1 mg/kg). Six months ago, the patient was readmitted to our hospital because of sudden dyspnea. Emergency chest CT and bronchoscopy showed granulation hyperplasia and scar tissue in the lumen of the subglottis to the upper segment of the tracheal stent, hyperemia and swelling of the mucosa, and a large amount of thick sputum blockage in the lumen, resulting in severe narrowing of the lumen (Figures 2R, 3Q). To avoid the risk of major airway bleeding and asphyxia during bronchoscopy under conventional ventilation, we decided to perform bronchoscopy-guided interventional therapy with VV-ECMO using a heparin-coated membrane lung.

**Figure 4:**
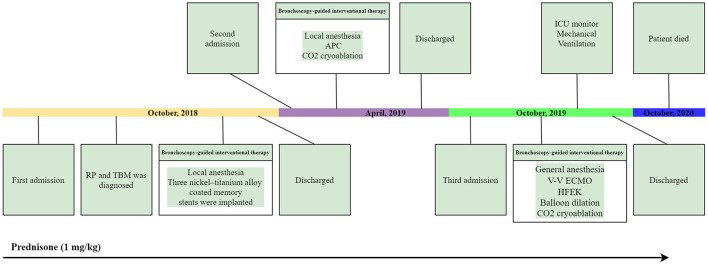
Clinical history of the patient. APC, argon plasma coagulation; HFEK, high-frequency electrosurgical knife.

We first percutaneously inserted a 22-Fr cannula into the left femoral vein and a 16-Fr venous cannula into the right internal jugular vein of the patient. The direction of the pipe connection was as follows: left femoral vein → centrifugal pump → membrane lung → right internal jugular vein. The circulatory system was pre-filled with Wanwen 1,500 mL and continuously infused with heparin during ECMO. The mean arterial pressure, SpO_2_, hematocrit, and activated clotting time (ACT) during transfusion were monitored. The ECMO speed was 3,500 rpm, the blood flow velocity was 3 L/min, the average arterial pressure was maintained at 90 ± 10 mmHg, and ACT was maintained at 250 s (4). We performed bronchoscopy interventional therapy under general anesthesia with oxygen supply guaranteed by ECMO. For the intervention, we first used a CO_2_ cryotherapy instrument to remove the local granulation tissue during the operation and then a needle-shaped high-frequency electrosurgical knife to make a radial cut on the narrow opening (Figure 3R). Subsequently, balloon dilations were performed three times at the lesion. Finally, CO_2_ cryoablation was performed (Figure 3S). The total treatment time was 1 h, the intraoperative bleeding volume was ~50 mL, and the SPO_2_ was maintained at 90–95%; the rest of the vital signs were stable. After the operation, the patient's tracheal stenosis significantly improved, and the bronchoscope was able to enter the distal airway smoothly, enabling the aspiration of large amounts of viscous secretions; the airway stent was in a good position and no serious complications, such as rupture were observed (Figures 3T–X, 2S,T). After the operation, the patient was transferred to the ICU for monitoring. After 12 h, ECMO support was stopped, and the patient was implanted with a laryngeal mask and switched to a mechanical ventilator for oxygenation. On the second day after surgery, we removed the laryngeal mask and switched to non-invasive ventilator-assisted ventilation. On the fourth day, the patient was discharged from the hospital. However, the patient eventually died due to sudden respiratory failure during half year follow-up. Clinical history of the patient can be seen in Figure 4.

## Discussion

RP is an immune disorder with an unknown etiology and multiple-system involvement. The literature shows that the disease mainly affects the cartilage tissues of the body, of which ear chondritis is the most common sign, usually manifesting as congestion, pain, swelling of the auricle cartilage and skin, and loss of normal auricle shape (5). The second is rhinochondritis, which often manifests as nasal congestion, pain, and even “saddle nose” deformity, but it rarely manifests as epistaxis (6). Moreover, the involvement of organs such as the heart, eyeballs, joints, skin, and nervous system are also common (7). RP usually occurs between 40 and 60 years of age, and fever, fatigue, weight loss, or skin rash may be the first symptoms (8). At least 50% of cases in the late stage of RP involve the airway, and both the upper and lower airway cartilage can be affected (9). Involvement of the larynx can cause stenosis of the glottis, manifested by hoarseness, wheezing, or tenderness in the front of the neck. The symptoms of tracheal and main bronchus involvement are often insidious, mainly manifested as TBM, which is a dynamic airway collapse and is a severe complication of RP (9); dry cough, dyspnea, and wheezing are the main symptoms. Because of disease progression and lack of effective treatments, it is obviously associated with a higher mortality rate.

The diagnosis of RP with TBM is often misdiagnosed as asthma (10). Once patients with RP have persistent cough, shortness of breath, and dyspnea, the possibility of TBM needs to be considered first. The gold standard for diagnosis is bronchoscopy, and the diagnostic criterion of TBM is reduction of the cross-sectional area of the trachea or bronchus lumen by at least 50% at the end of expiration or coughing compared with the inspiratory phase (2, 3). Considering that bronchoscopy is an invasive examination, chest CT has recently been recommended as an alternative method, and it has good sensitivity and specificity (11). Chest CT examination requires a biphasic CT scan, including the inspiratory and expiratory phases, and the diagnostic criteria are consistent with bronchoscopy (12). Spirometry also plays an important role in the diagnosis of TBM. The flow volume curve is characterized by a decrease in the flow rate from the peak flow to an inflection point with a peak flow rate <50%. The inflection point occurs within the first 25% of the expired vital capacity. The inspiratory limb of the curve showing no evidence of obstruction was observed in almost all patients (13). Spirometry in patients with TBM may reveal obstructive ventilatory impairment but does not correlate with the severity of airway narrowing (14). Besides, studies have shown that PET-CT also has a better effect in diagnosing TBM (15); however, the cost is too high, which is not conducive to general screening of the disease.

Currently, glucocorticoids and methotrexate are the most important drugs for the treatment of RP, and long-term use can prevent further deterioration of TBM. Nevertheless, there are still many reports on treatment failure. Non-invasive ventilators can provide continuous positive airway pressure, help maintain airway patency, and have a certain effect on patients with mild TBM (2, 3). Recently, reports of airway stents, including metal stents, silicone stents, and Montgomery T-tubes, used in RP with TBM have increased gradually (16–18). Studies have shown that stents can maintain airway stability and significantly improve airway collapse and its consequent symptoms (19). However, long-term follow-ups and prognostic data are generally lacking. In addition, long-term airway stent implantation also has many complications, such as displacement, fracture, granulation hyperplasia, airway bleeding, and mucus obstruction, which may affect the efficacy and subsequent treatment (20–22). The literature shows that tracheobronchoplasty has a better effect on severe TBM caused by COPD and can significantly improve recent clinical symptoms and quality of life (19). However, this therapy often requires surgical intervention, which is more traumatic and has more complications, including post-operative death. The effect of tracheobronchoplasty on TBM caused by RP has not yet been reported. Therefore, the treatment of RP with TBM remains challenging. For RP with severe TBM with respiratory failure or cardiac insufficiency, conventional mechanical ventilation and general anesthesia often cannot guarantee the oxygen supply or safety during the operation. Hence, ECMO as an alternative for cardiopulmonary function, plays an important role in bronchoscopy-guided interventional therapy.

ECMO is also called extracorporeal life support. Its main purpose is to provide blood oxygenation, remove carbon dioxide, and ensure effective blood supply to the body; hence, by providing emergency and critically ill patients with respiratory and circulatory support, thereby playing an important role in emergency and critical care (4). The treatment modes of ECMO mainly include VV-ECMO and venous-arterial ECMO (VA-ECMO). The former is mainly used for respiratory failure and ARDS, and the latter is mainly used for cardiac surgery. In addition, VA-ECMO used in Extracorporeal Cardio-Pulmonary Resuscitation (E-CPR) can improve survival with good neurologic outcomes when initiated early in selected patients (23). Recently, ECMO has been gradually used in bronchoscopy-guided interventional therapies. For example, Natt et al. successfully performed balloon dilatation and tracheal stenting with VV-ECMO support in patients with severe tracheal occlusion after tracheal intubation, and the patient's post-operative dyspnea was significantly restored (24). With the support of ECMO, Kim et al. successfully performed bronchoscope-guided tumor resection in an 88-year-old patient with tracheal metastases of a mediastinal teratoma (25). Although reports of ECMO used in RP with TBM are rare, they have shown important clinical value [(26–29); Table 1]. Mitilian et al. reported a case of severe RP with TBM, who developed extensive airway tear, bilateral pneumothorax, and mediastinal emphysema after a Y-stent was placed under general anesthesia. After failure of mechanical ventilation, the patient was successfully discharged from the hospital with the help of VV-ECMO (28). Laliberte et al. reported that a patient with RP and severe TBM had tracheal perforation and subcutaneous emphysema when the Dumon silicone stent was replaced, but the airway was successfully repaired after reinserting the Y-shaped silicone stent with the assistance of VA-ECMO (29). Although ECMO provides adequate cardiopulmonary support, it also has complications such as hemorrhage, embolism, hemolysis, edema, and infection (30). Sy et al. conducted a systematic review of the complications of ~1,496 patients in 26 studies using ECMO. The results showed that bleeding was the most common complication of ECMO, with a prevalence rate of 27%, and the overall prevalence of thromboembolic events was 8%. Among them, limb ischemia, blood vessel-related coagulation, and stroke are the most frequently reported events (31). We successfully performed a bronchoscopy-guided intervention therapy with ECMO support for advanced cancer metastasis to the central airway, and the tumor was completely removed by surgery. Although airway oozing and blood clots filled part of the bronchus after the operation, no other complications occurred after adjusting the heparin dose and airway clearing (4). Although this patient reported in this article eventually died, the specific reasons are complicated. In addition to RP and TBM, COPD and lung bullae can also cause respiratory failure. Moreover, the irreversible progression of TBM and RP and stent-related complications can also aggravate the original symptoms and disease risks. Despite the aforementioned shortcomings of ECMO, its important role in bronchoscopy-guided interventional therapy is very obvious: first, even though the intervention and anesthesia share the airway, ECMO eliminates the interference of tracheal intubation, providing a more open and clear surgical field; second, it allows the surgeon a longer operation time and more room to perform the surgery in an orderly manner; finally, it maintains stable oxygenation and hemodynamics during surgery (4). However, hemorrhage commonly occurs during bronchoscopy-guided therapy, and systemic heparinization during ECMO is bound to further increase the risk of coagulopathy, such as major bleeding and embolism. Therefore, further research on the amount and timing of heparin should be conducted in the future to improve the safety of interventional surgery. Besides, the practices of ECMO and bronchoscopy-guided intervention therapy need adequate technical skills that can be acquired only through defined learning pathways (32). The case in this article shows that VV-ECMO can provide sufficient oxygenation and safety for bronchoscopy-guided interventional therapy for RP with TBM.

**Table 1:** The reported literature of ECMO used in RP with TBM.

**ECMO treatment of RP involving the airway**									
**Num**.	**Age**	**Gender**	**Main symptoms**	**Primary diagnosis**	**Initial treatment**	**Complications**	**Later treatment**	**Follow-up**	**References**
Case 1	24	Man	Dyspnea	RP and airway severe obstruction	Montgomery T-tube	Tracheal tear; Bilateral Tension pneumothorax; Tension pneumoperitoneum	VV-ECMO + tracheotomy	Death	(25)
Case 2	39	Man	Dyspnea	RP and airway stenosis	Dumon stent	Laceration of trachea and left main stem bronchus; Acute respiratory distress; Subcutaneous emphysema	PCPS (like ECMO) + esophageal tracheobronchoplasty	Good	(26)
Case 3	41	Man	Dyspnea	RP and airway malacia	Y stent	Perforation of the tracheal membranous; Bilateral tension pneumothorax; Hemodynamic instability	VV-ECMO + tracheotomy	Good	(27)
Case 4	55	Man	Dyspnea	RP and tracheomalacia	Dumon stent	Tracheal tear; Subcutaneous emphysema	VA-ECMO + silicone Y stent	Good	(28)

*ECMO, extracorporeal membrane oxygenation; RP, relapsing polychondritis; PCPS, percutaneous cardiopulmonary support*.

## Conclusion

TBM is a common and serious complication of RP involving the airway. ECMO can be used as an important support tool for patients with cardiopulmonary insufficiency or severe airway stenosis when conventional general anesthesia and mechanical ventilation cannot maintain oxygenation or ensure safety during bronchoscopy-guided intervention therapy.

## Data Availability Statement

The original contributions presented in the study are included in the article/supplementary material, further inquiries can be directed to the corresponding author/s.

## Ethics Statement

Written informed consent was obtained from the individual(s) for the publication of any potentially identifiable images or data included in this article.

## Author Contributions

PZ and WY: conception and design. QX and CZ: administrative support. KC and WT: provision of study materials or patients. WY and BF: collection and assembly of data. PZ and BF: data analysis and interpretation. PZ, BF, and WY: manuscript writing. WY and WH: final approval of manuscript. All authors contributed to the article and approved the submitted version.

## Funding

This work was funded by the 2020 Xinglin Scholars Scientific Research Promotion Plan of Chengdu University of Traditional Chinese Medicine (QNXZ2020007) and the Hundred Talents Plan Project of Hospital of Chengdu University of Traditional Chinese Medicine (20-Q07). The funder does not take part in the study design, data collection, and analysis, or the preparation of the manuscript. The funder has provided only financial support for the study.

## Conflict of Interest

The authors declare that the research was conducted in the absence of any commercial or financial relationships that could be construed as a potential conflict of interest.

## Publisher's Note

All claims expressed in this article are solely those of the authors and do not necessarily represent those of their affiliated organizations, or those of the publisher, the editors and the reviewers. Any product that may be evaluated in this article, or claim that may be made by its manufacturer, is not guaranteed or endorsed by the publisher.

## Abbreviations

CT: computed tomography
ECMO: extracorporeal membrane oxygenation
RP: relapsing polychondritis
TBM: tracheobronchomalacia
ACT: activated clotting time.
